# Subcapsular hepatic hematoma as a complication of severe preeclampsia: a case report

**DOI:** 10.1186/s13256-021-03166-w

**Published:** 2021-12-17

**Authors:** Kiel Luhning, Hilary MacCormick, Bruce Macaulay, Marianna Saunders, Catherine Craig

**Affiliations:** Dalhousie University, Halifax, NS Canada

**Keywords:** Severe preeclampsia, Subcapsular (liver) hematoma, Unruptured hematoma, Infected pleural effusion, Video-assisted thoracoscopic surgery

## Abstract

**Background:**

Subcapsular hepatic hematoma is a rare and life-threatening complication of pregnancy. It is most commonly associated with severe preeclampsia and hemolytic anemia, elevated liver enzymes, and low platelets syndrome. Patients with subcapsular hepatic hematoma typically present with epigastric, right upper quadrant or shoulder pain, nausea and vomiting, and/or shortness of breath. Here we describe a patient with a classic pain presentation, a large unruptured hematoma, and an unusual postpartum course.

**Case:**

A 40-year-old gravida 1 para 0 Caucasian woman presented at 39 + 6 weeks gestational age with a 3-day history of new onset pain in an otherwise uncomplicated pregnancy. She described the pain along her right torso as severe, shooting, and sharp, but at times pleuritic in nature. She was found to have new onset preeclampsia and hemolytic anemia, elevated liver enzymes, and low platelets syndrome. Induction of labor was initiated and eventually she delivered by cesarean section. Her pain persisted in the postpartum period and abdominal computed tomography scan revealed a 16 cm subcapsular hepatic hematoma. Despite the hematoma being thin walled, conservative management was recommended by the general surgeon. She then re-presented on postpartum day 15 with tachypnea, dyspnea, and pleuritic chest pain. Secondary to the subcapsular hepatic hematoma, she then developed an infected and loculated, large pleural effusion. This required video-assisted thoracoscopic surgery before her eventual discharge home on postpartum day 21.

**Conclusions:**

There should be high clinical suspicion of subcapsular hepatic hematoma in patients with persistent pain in the right upper quadrant of the abdomen. Urgent imaging to investigate for subcapsular hepatic hematoma is then indicated. Cesarean delivery without labor and treatment for severe preeclampsia should be undertaken if subcapsular hepatic hematoma is found. Conservative management and serial imaging are reasonable for the follow-up of a large, unruptured hematoma. Hepatic artery embolization should also be considered. Subcapsular hepatic hematoma may be complicated by infected pleural effusions and require video-assisted thoracoscopic surgery.

## Background

Subcapsular hepatic hematoma (SCH) is a rare and life-threatening complication of pregnancy [1]. The pathogenesis of SCH is not well understood. The common proposed mechanism includes endothelial injury secondary to preeclampsia, fibrin deposition, hepatic sinusoidal obstruction, neovascularization, and microhemorrhage leading to hematoma formation [2–5]. Rarely, SCH are reported with coexisting pleural effusion or renal hematoma [3]. SCH is most commonly associated with severe preeclampsia and hemolytic anemia, elevated liver enzymes, and low platelets (HELLP) syndrome [1]. However, the differential diagnosis includes hepatic neoplasms, infectious processes, aneurysms, and biliary disease [6]. Although preeclampsia occurs more commonly in young primigravida, SCH seems to be more common in multiparous patients with advanced maternal age [5]. Unfortunately, there is no correlation between laboratory abnormalities and presence of SCH [2]. However, the diagnosis of SCH must be considered in patients with stereotypical pain presentation, including severe shoulder, back, or epigastric pain, particularly if the pain does not respond to conservative treatments, including acetaminophen, opioids, and epidural analgesia [2, 3, 6, 7]. Here we describe a patient with a classic pain presentation, a large unruptured hematoma, and an unusual postpartum course.

## Case

A 40-year-old gravida 1 para 0 Caucasian woman presented at 39 + 6 weeks gestational age with a 3-day history of new onset pain in an otherwise uncomplicated pregnancy. At 39 + 1 weeks gestation she had started outpatient cervical ripening with dinoprostone (Cervidil) because of her advanced maternal age. She described the pain along her right torso as severe, shooting, and sharp, but at times pleuritic in nature. It rapidly progressed from her right trapezius to encompass the entirety of her right torso, from her upper abdominal quadrant and epigastrium, radiating to her back, chest, shoulder, and neck. Her pain was initially attributed to possible radiculopathy, as she had a previous history of the same. Her vital signs at initial presentation were normal, with no hypertension noted, and there were no concerns regarding the fetal status. No further investigations were ordered and a full neurological examination was not documented.

She represented less than 24 hours later at 40 + 0 weeks gestational age, and was found to be hypertensive at 157/101 and 164/112 mmHg. Oxygen saturation was 98% on room air. She had sinus tachycardia on arrival, ranging from 110 to 140 bpm and persisting throughout the peripartum period. She was tender in the right upper quadrant, however there were no peritoneal signs, and no hepatomegaly was appreciated in the presence of the gravid uterus. She was tender from the right side of her neck through to her right lower back. Her reflexes were 3 + bilaterally with no clonus. The fetal heart rate was normal. Her blood investigations revealed a hemoglobin of 105 g/L, platelets 156,000 g/L, alanine aminotransferase (ALT) 193 μ/L, aspartate aminotransferase (AST) 111 μ/L, and uric acid 429 μmol/L. White blood cell count, creatinine, lactate dehydrogenase (LDH), and coagulation profile were within the normal range. An electrocardiogram (ECG) showed sinus rhythm with no abnormal features, apart from the previously noted tachycardia.

After diagnosing preeclampsia, induction of labor was started, as was an infusion of magnesium sulfate. Due to the unusual but significant pain that she was experiencing, a range of differential diagnoses were considered by the obstetrical and anesthesia team. Preeclampsia with HELLP syndrome was the working diagnosis, however differential diagnoses included fatty liver, radiculopathy, cholecystitis, pancreatitis, and pyelonephritis. The patient requested labor analgesia be initiated and a combined spinal epidural was placed when the patient’s cervix was 2 cm dilated. Analgesia was maintained via programmed intermittent epidural bolus with patient-controlled epidural analgesia as needed, in keeping with our institutional practice. The patient reported satisfactory analgesia with regard to her labor pain, but still complained of severe pain in her right torso that was unresponsive to acetaminophen and opioids. Blood investigations monitored every 4 hours remained stable with hemoglobin of 103–102 g/L, platelets 158,000–165,000 g/L, ALT 176–169 μ/L, AST 100–97 μ/L, and uric acid 400–420 μmol/L. The LDH, creatinine, and coagulation profile remained normal. Labor progressed to full dilation with oxytocin augmentation, but as a result of torso pain she was unable to exert adequate effort with pushing. Ultimately, the obstetrical team consented the patient for a trial of forceps and possible cesarean delivery.

**Fig. 1:**
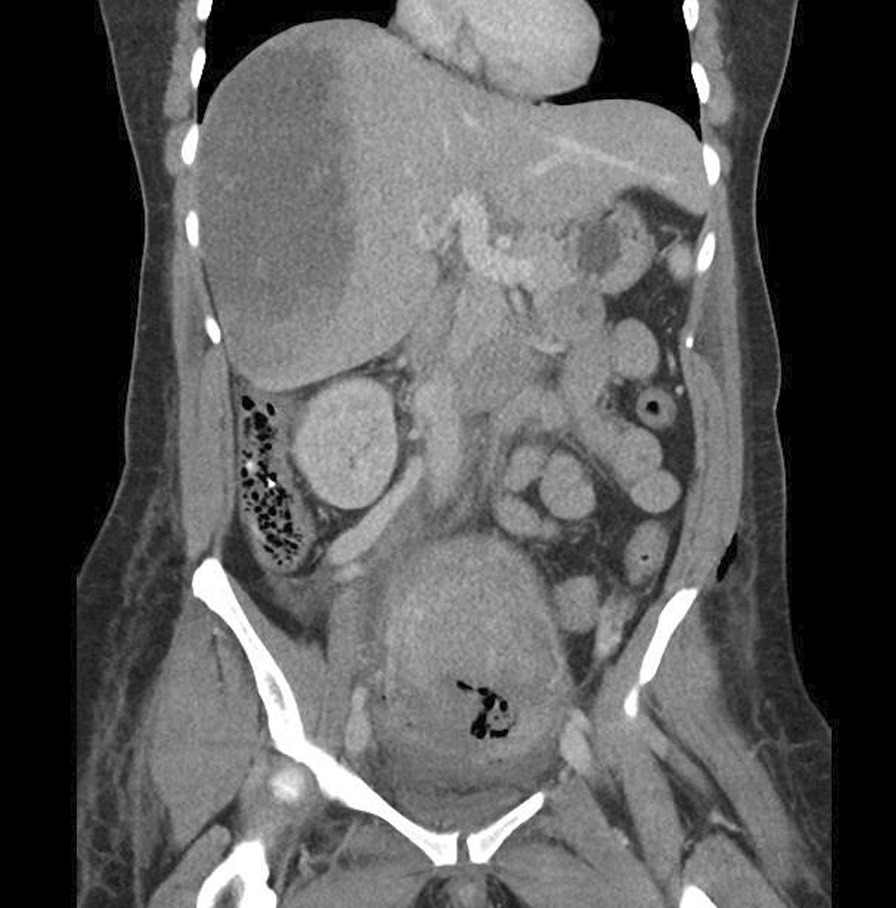
1. Initial CT abdomen: Large subcapsular liver hematoma measuring 16 cm in maximum cranial-caudal dimension. Heterogeneous attenuation is compatible with blood product of varying ages. No active bleed was identified. There is significant mass effect on the liver, however the liver parenchyma was normal.

In the operating room, the obstetrics team performed an examination under anesthesia, which revealed the fetus to be in an occiput-transverse position at station zero, and the decision was made to proceed to cesarean delivery as the station was too high to perform a trial of forceps. A nonvigorous male infant was delivered with Apgar scores of 3 and 8 at 1 and 5 minutes, respectively. Placental delivery was uneventful. Atonic postpartum hemorrhage was treated with a bolus of 2 units IV oxytocin and carboprost 250 mcg intramuscular (IM), in addition to an oxytocin infusion. As per our institutional practice, a bolus of oxytocin is only used when the patient has multiple risk factors for intraoperative hemorrhage or is experiencing uterine atony. This low dose was chosen to prevent potential side effects (nausea, vomiting, chest pain) and complications (hypotension, hypertension, ST segment changes, myocardial ischemia, bronchospasm) that can be associated with higher dose boluses when given at the time of cesarean section [8, 9]. Prior to fascial closure, the obstetrics team manually explored the abdomen to the extent possible through the Pfannenstiel incision. No gross abnormalities were identified. The patient’s right torso pain persisted throughout her time in the operating room, with only a slight improvement after surgical anesthesia obtained via the epidural. After delivery, 2.5 mg of epidural morphine was given for postoperative analgesia. The patient was hemodynamically stable throughout the operation, although remained tachycardic. Estimated blood loss was approximately 900 mL. Postoperatively, the patient was sent for a computed tomography (CT) scan of her chest, abdomen, and pelvis, including contrast for a pulmonary embolism protocol to investigate the abdominal pain and persistent tachycardia. No pulmonary embolus was found and a small right pleural effusion with subsegmental atelectasis was noted. However, the most impressive feature on the scan was a large but intact subcapsular liver hematoma that measured approximately 16 × 7 × 14 cm (Fig. 1). Portal hypertension was suggested by the presence of portosystemic varices. The general surgery team was consulted and recommended conservative management with strict blood pressure control requiring oral labetalol, frequent complete blood count (CBC) monitoring, and 48 hours of bedrest. If, however, the SCH had been diagnosed antepartum, labor would have been avoided and delivery would have been expedited via immediate cesarean section. The SCH is at risk of rupture with active pushing, convulsions, or abdominal trauma, including vigorous palpation of the right upper quadrant [2]. In this case, prophylactic antibiotics were not used as there were no signs of infection and the underlying etiology was noninfectious. Interventional radiology was on standby to perform hepatic artery embolization if the SCH significantly increased in size or there was concern about imminent rupture. Serial monitoring of blood work revealed a hemoglobin of 70 g/L and 56 g/L on postoperative days 1 and 2, respectively, prompting transfusion of two units of packed red blood cells on day 2. The patient was discharged on postoperative day 5 with her hemoglobin stable at 85 g/L.

**Fig. 2:**
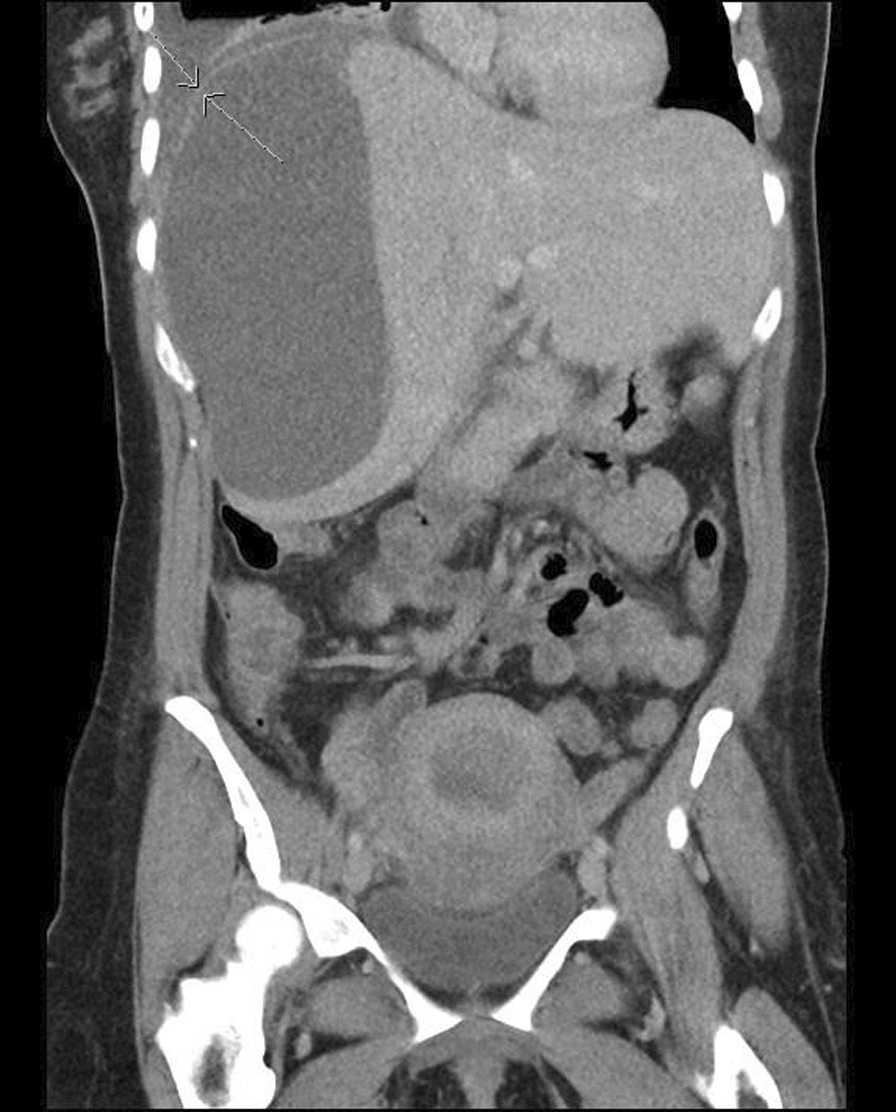
Computed tomography abdomen postoperative day 15: Large subcapsular liver hematoma has increased in size, now measuring 18 cm in cranial-caudal dimension. However, no CT features of active bleed are present. Along the superior margin of the hematoma, the liver capsule demonstrates marked thinning.

**Fig. 3:**
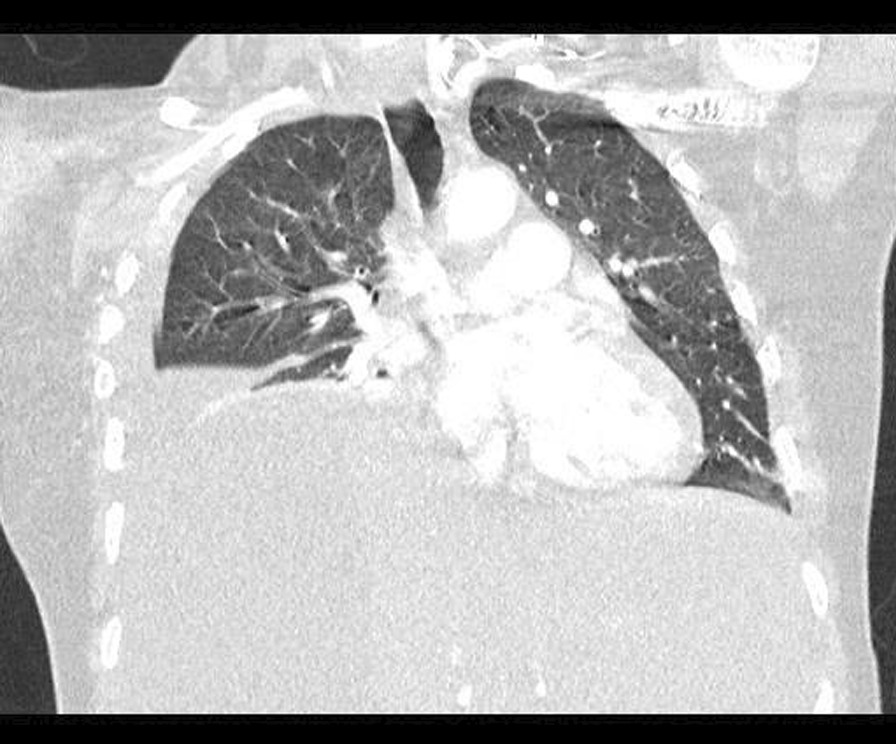
Computed tomography chest postoperative day 15: Large right sided pleural effusion with mediastinal shift.

An early postoperative follow-up visit was arranged 15 days postpartum and at that visit the patient was noted to be pale, tachypneic, and upon questioning she stated increasing shortness of breath and a persistence of her pleuritic chest pain. She was sent back to the IWK Health Centre where her vital signs were as follows: heart rate 110–130 bpm, blood pressure 112/66 mmHg, oxygen saturation 97% on room air, respiratory rate 40–48, temperature 37.9 °C. Blood investigations revealed a white cell count of 6, hemoglobin 97 g/L, platelets 650,000 g/L, ALT 75 μ/L, AST 88 μ/L, LDH 1310 μ/L, and coagulation profile remained normal. A repeat abdominal CT scan reported an increase in the size of the liver hematoma to 14 × 8.5 × 18.3 cm (Fig. 2). Although there was no evidence of active intralesional bleeding or rupture, the liver capsule was difficult to visualize in the superior aspect, and could suggest significant thinning and imminent rupture. The CT scan of the chest reported a large right pleural effusion with mediastinal shift (Fig. 3).

The patient was urgently transferred from the stand-alone maternity hospital to a nearby hospital under the care of the thoracic surgery team, with the general surgery team on standby. A chest tube was placed, but ultimately the patient required video-assisted thoracoscopic surgery (VATS), with right partial decortication of an infected, loculated pleural effusion prior to her discharge home. The pleural effusion aspirate showed no growth. The right pleural effusion was felt to be exudative in nature and secondary to the adjacent large SCH. Throughout the admission she remained normotensive and her hepatic hematoma remained stable with no signs of active bleeding. She was discharged home on day 2 following the VATS procedure, coinciding with day 21 following her cesarean section. At discharge, her hemoglobin was 74 g/L and her platelets were 884,000 g/L.

She continued to receive follow-up with general surgery and obstetrics as an outpatient. A CT performed 4 weeks postpartum showed the hepatic hematoma had decreased in size to 12.6 × 6.4 × 15.1 cm and at 6 months postpartum had decreased to 3.8 × 2.2 × 3.1 cm and did not require any further follow-up.

## Conclusion

SCH should be suspected in patients who present with severe preeclampsia or HELLP syndrome, particularly if the patient’s right upper quadrant or epigastric pain is unrelenting and unresponsive to conventional interventions such as acetaminophen, opioids, and epidural analgesia. Timely diagnosis is critical as this can be a life-threatening illness, especially if the SCH ruptures [1]. Investigation with either abdominal ultrasound, computed tomography, or magnetic resonance imaging accurately diagnoses the presence of SCH [1, 3]. If diagnosed antepartum, it is best to treat for severe preeclampsia with infusion of magnesium sulfate, blood pressure control, and expedited delivery via immediate caesarean section [6]. If SCH is diagnosed postpartum, conservative management is appropriate if the patient is clinically stable [1, 2, 5–7, 10]. Serial imaging and strict blood pressure control are warranted. Hepatic artery embolization should also be considered if local expertise is available. One should also bear in mind that patients with SCH can also rarely develop large, infected pleural effusions that may require a VATS procedure, as seen in this patient.

## Data Availability

Not applicable.

## Abbreviations

SCH: Subcapsular hepatic hematoma
HELLP: Hemolytic anemia, elevated liver enzymes, and low platelets
VATS: Video-assisted thoracoscopic surgery
